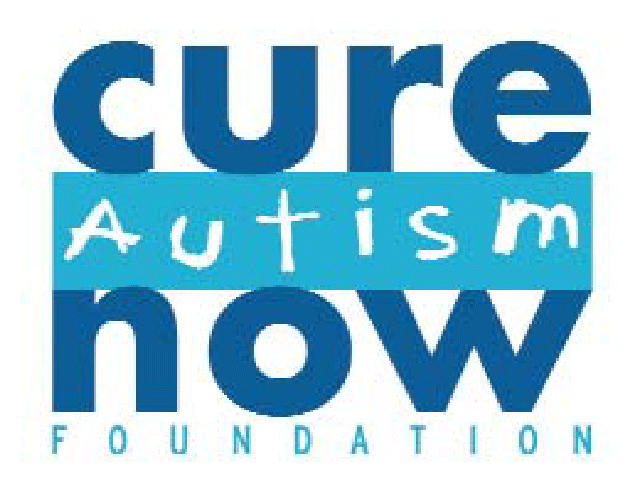# EHPnet: Cure Autism Now

**Published:** 2006-07

**Authors:** Erin E. Dooley

In 1995, parents of children with autism joined together to form the nonprofit organization Cure Autism Now (CAN). Since then, its membership has grown to include clinicians and scientists committed to accelerating the pace of biomedical research in autism. CAN raises and distributes funds for research on the causes, prevention, and treatment of autism, as well as education and outreach. As a resource for everyone interested its work, CAN has a website located at **http://www.cureautismnow.org/**.

So far, CAN has committed over $25 million to research funding and has established and continues to support the Autism Genetic Resource Exchange (AGRE). Clicking on the Research link at the top of the CAN homepage takes visitors to an overview of the CAN science program, which includes six initiatives that the group believes will yield the most effective treatment for individuals with autism.

The Genomics Initiative focuses on gene mapping and microarray work. CAN’s goal is to identify several genes involved in autism within the next three years. Closely related to the Genomics Initiative is the AGRE, an open gene bank with a large collection of immortalized cell lines and DNA samples gathered from families with more than one autistic child. Available on the AGRE page is a link to research updates published since 2001.

The goal of the Innovative Technology for Autism Initiative is to stimulate development of products that provide realistic solutions to the issues encountered by those with autism, their families, educators, health care specialists, and researchers. The initiative offers multiyear grants, fast-track “bridge” grants, and educational programs. It also sponsors a workgroup within which investigators can meet, share, and collaborate, and which also serves to actively bring new investigators into the field.

One major hurdle that autism researchers are working to overcome is the lack of any biomarker for diagnosis. The CAN Biomarkers Initiative has yielded two preliminary findings of possible autism bio-markers—one a novel protein in the urine of children with autism and some of their unaffected relatives, and the other a distinct lipid profile that was seen in 20 AGRE samples. CAN has launched a study in an effort to replicate and confirm these results.

In the past few years, new findings on neuroplasticity, the ability of the brain to grow and change throughout life, have led to significant breakthroughs in the treatment of stroke and dyslexia through a process called neural retraining. To apply these same ideas to the treatment of autism, CAN has established the Neural Retraining Initiative. The initiative’s first project, led by Michael Merzenich of the University of California, San Francisco, is working to design, produce, and test nonpharmaceutical tools and techniques, including one to prevent the emergence of full-blown autism in at-risk infants.

CAN has also awarded several grants through its Environmental Factors in Autism initiative to study the neurotoxicity of mercury and how it may factor in the development of autism. Thimerosal, which contains ethylmercury, has been widely used as a preservative in vaccines and other health and medical products, and has been raised as a potential contributor to autism.

## Figures and Tables

**Figure:**